# Increased risk of abortion after frozen-thawed embryo transfer in women with polycystic ovary syndrome phenotypes A and D

**DOI:** 10.1038/s41598-022-18704-9

**Published:** 2022-09-01

**Authors:** Qiumin Wang, Yanjun Zheng, Ping Li, Guanqun Zhang, Shanshan Gao, Ze Wang, Baozhen Hao, Yuhua Shi

**Affiliations:** 1 Center for Reproductive Medicine, Shandong University, 44 Wenhuaxi Road, Jinan, 250012 China; 2 Key Laboratory of Reproductive Endocrinology of Ministry of Education, Shandong University, Jinan, 250012 Shandong China; 3 Shandong Key Laboratory of Reproductive Medicine, Jinan, 250012 Shandong China; 4 Shandong Provincial Clinical Research Center for Reproductive Health, Shandong University, 44 Wenhuaxi Road, Jinan, 250012 China; 5 National Research Center for Assisted Reproductive Technology and Reproductive Genetics, Shandong University, Jinan, 250012 Shandong China; 6 Women and Children’s Hospital, School of Medicine, Xiamen University, 10 Zhenhai Road, Xiamen, 361003 China; 7 Shandong Provincial Maternal and Child Health Care Hospital, Cheeloo College of Medicine, Shandong University, Jinan, China

**Keywords:** Endocrinology, Medical research, Risk factors, Reproductive disorders

## Abstract

Polycystic ovary syndrome (PCOS) is associated with adverse pregnancy outcomes, including an increased risk of abortion, premature delivery, and even neonatal outcomes. After removing the effect of COH on patients, studying the pregnancy outcomes of patients with different PCOS phenotypes after FET may better reflect the impact of different PCOS phenotypes on ART outcomes. Data of 8903 patients who underwent FET between January 2017 and October 2019 were retrospectively collected and evaluated. All patients were divided into a control group and four phenotype groups based on Rotterdam criteria. The main outcomes were pregnancy outcomes after FET. We found significantly higher abortion (*P* = 0.010) and lower ongoing pregnancy (*P* = 0.023) rates for women with PCOS phenotypes A and D compared to those in the control group. After adjusting for potential confounders, PCOS phenotypes A and D were associated with an elevated risk of abortion (adjusted OR, 1.476, *P* = 0.016; adjusted OR, 1.348, *P* = 0.008, respectively). The results of this study suggest that when performing FET, clinicians should individually manage women with PCOS phenotypes A and D to reduce the rate of abortion and increase the rate of LB, and achieve better pregnancy outcomes.

## Introduction

Polycystic ovary syndrome (PCOS) is considered a common endocrine disorder in women of reproductive age^1^, affecting 6–21% of women worldwide^2^. Characteristics of PCOS include obesity, insulin resistance, hyperandrogenism, anovulation, and polycystic ovaries^3^. Due to endocrine disorders and anovulation in women with PCOS, which lead to infertility^4^, such women usually require assisted reproductive technology (ART) to become pregnant.

Applying PCOS diagnostic criteria, four phenotypes are distinguished: phenotype A: coexistence of clinical hyperandrogenism/hyperandrogenemia (HA), oligomenorrhea/anovulation (OA), and polycystic ovary morphology (PCOM); phenotype B: HA and OA without PCOM; phenotype C: HA and PCOM with regular ovulatory cycles; and phenotype D: OA coexisting with PCOM^2^. Patients with different PCOS phenotypes show different ovarian responses to controlled ovarian hyperstimulation (COH)^5^, which might contribute to different pregnancy outcomes^6^. The previously published literature has mainly described pregnancy outcomes in patients with different PCOS phenotypes after fresh embryo transfer^6,7^; patients with PCOS are prone to ovarian hyperstimulation syndrome (OHSS) during or after COH^8^. Clinically, a fresh embryo transfer is usually canceled to reduce the risk of OHSS^9^. However, it has been reported that frozen-thawed embryo transfer (FET) can not only reduce the risk of OHSS but also improve ART outcomes^10^. Recently, a multicenter randomized controlled trial of infertile women with PCOS suggested FET led to a higher rate of live births, and a lower risk of abortion than did fresh embryo transfer^11^. FET allows time for the use of preimplantation genetic technology^12^. Therefore, a considerable number of infertile patients choose to undergo FET. However, PCOS is associated with adverse pregnancy outcomes, including an increased risk of abortion, premature delivery, pre-eclampsia, and even neonatal outcomes^13,14^. After removing the effect of COH on patients, studying the pregnancy outcomes of patients with different PCOS phenotypes after FET may better reflect the impact of different PCOS phenotypes on ART outcomes. Subsequent clinical management, which might optimize pregnancy outcomes, could then be carried out based on different PCOS phenotypes.

This study aimed to assess the effect of various PCOS phenotypes after FET on pregnancy outcomes.

## Materials and methods

### Study design and patients

This study retrospectively collected and evaluated the clinical data of 8903 patients after FET at the Center for Reproductive Medicine, Cheeloo College of Medicine, Shandong University, who were patients between January 2017 and October 2019. All women between the ages of 20 to 40 years with a body mass index (BMI) of no more than 35 kg/m2, underwent their first FET during their first in vitro fertilization/intracytoplasmic sperm injection cycle at our center. Informed consent was obtained from all patients in this study.

All women were excluded from this study if they (i) underwent frozen-thawed oocyte cycle or preimplantation genetic testing cycles, (ii) were diagnosed with premature ovarian insufficiency or a decreased ovarian reserve, (iii) had a history of unilateral oophorectomy, recurrent spontaneous abortion or severe intrauterine adhesion, (iv) had medical conditions that contraindicated assisted reproductive technology or pregnancy, and (v) were diagnosed with hypertension, diabetes, abnormal renal function, uterine malformation or abnormal parental karyotypes.

### Ethics approval and consent to participate

All procedures performed in studies involving human participants were following the ethical standards of the institutional and/or national research committee and with the 1964 Helsinki declaration and its later amendments or comparable ethical standards. The institutional review board of the Center for Reproductive Medicine, Cheeloo College of Medicine, Shandong University approved this study (2021-25), and all research was performed by relevant guidelines/regulations. Informed consent was obtained from all patients.

All women were assigned to either PCOS (n = 1887) or control (n = 7016, women without PCOS, and the above-mentioned inclusion and exclusion criteria were also used to screen the control subjects.) groups. A diagnosis of PCOS was based on Rotterdam diagnostic criteria and if at least two of the following criteria were present: HA (hyperandrogenemia, defined as total testosterone levels above 48.1 ng/dL, and hirsutism with a total score ≥ 8 according to the Ferryman–Gallwey score); OA (defined as a delay of > 35 days or < eight spontaneous hemorrhagic episodes/years); PCOM (defined as ≥ 12 small follicles measuring 2–9 mm in at least one ovary or ovarian volume ≥ 10 cm^3^)^15^. Patients with PCOS were categorized into four phenotype groups according to Rotterdam criteria as follows^16^: phenotype A group (n = 452): HA + OA + PCOM; phenotype B group (n = 88): HA + OA; phenotype C group (n = 119): HA + PCOM; and phenotype D group (n = 1228): OA + PCOM.

### Measurement

Patient data were obtained from our center records, including age, BMI, systolic blood pressure (SBP), diastolic blood pressure (DBP), duration and type of infertility, past medical history, a basic vaginal ultrasound, a basal hormone profile evaluation, and outcomes of COH, as well as laboratory and clinical features of FET cycles. Blood samples of a basal hormone profile were collected for assessment on days 2–5 of a spontaneous or progestin-induced menstrual cycle in all women. All the hormonal assays were performed in the laboratories of the Center for Reproductive Medicine, Cheeloo College of Medicine, Shandong University.

### Treatment

All patients received a standard ovarian stimulation protocol (the antagonist or the long protocols), oocyte retrieval, fertilization, embryo cultured and cryopreserved in vitro, and luteal phase support protocol after embryo transfer (ET), according to a routine method^17^. All the FET patients in this study were not suitable for fresh embryo transfer or did not get a viable neonate after fresh embryo transfer. The outpatient physician chose the appropriate protocol based on clinical indications to prepare endometrium and mainly included a natural cycle, hormonal replacement therapy cycle, and ovulation induction cycle. Frozen blastocysts were thawed and transferred, and subsequently provided luteal phase support protocol according to the different endometrial preparation programs^18,19^.

### Outcomes of FET

The primary outcomes after FET were pregnancy outcomes consisting of biochemical pregnancy, clinical pregnancy (CP), ectopic pregnancy, abortion, premature delivery, and live birth (LB). Biochemical pregnancy was indicated by a serum human chorionic gonadotropin (HCG) level ≥ 10 IU/L on day 14 after ET. Clinical pregnancy was considered to detect the presence of a gestational sac by ultrasonography on day 35 after ET. A diagnosis of ectopic pregnancy occurred when a developing blastocyst was found implanted outside the endometrial cavity. Abortion was defined as a clinical pregnancy lost before 28 weeks gestation. Premature delivery was regarded as neonatal birth from the 28th to the 37th week of gestation. Live birth was considered as the delivery of any viable neonate at 28 weeks of gestation or later.

### Statistical analyses

Group means were compared using a one-way analysis of variance (One-Way ANOVA or Welch’s ANOVA was based on the results of the variance homogeneity test (Levene test). LSD (equal variances assumed) or Games-Howell (equal variances not assumed) were used for Post hoc multiple comparisons.) for quantitative variables, and a chi-squared test for qualitative variables. Quantitative variables were expressed as the mean ± standard deviation (normal distribution or near-normal distribution, Data were tested for normality by the Shapiro–Wilk normality test), and qualitative variables were presented as frequencies and percentages. Multivariate logistic regression analysis (backward: conditional) was performed to compare adjusted odds ratios (OR) and 95% confidence intervals (CI) for the effect of various PCOS phenotypes on pregnancy outcomes in FET, adjusted by the variates of *P* < 0.05 in univariate logistic regression analysis.

All analyses were performed with the use of SPSS software (version 23.0). Two-sided *P* values of less than 0.05 were considered to indicate statistically significant differences.

## Results

### Patient’s characteristics and outcomes of COH

A total of 8903 patients who underwent FET were enrolled in the present study and categorized as 1887 PCOS women and 7016 non-PCOS women. Of the 1887 women with PCOS, 452 showed phenotype A (HA + OA + PCOM, 23.95% of PCOS), 88 presented with phenotype B (HA + OA, 4.66% of PCOS), 119 revealed phenotype C (HA + PCOM, 6.31% of PCOS), and 1228 displayed phenotype D (OA + PCOM, 65.08% of PCOS), that may be related to the endocrine characteristics of the Chinese women. In addition, of the 7016 non-PCOS women in the control group, 218 showed HA (3.10% of non-PCOS women), 868 showed OA (12.40% of non-PCOS women), 1091 showed OA (15.60% of non-PCOS women).

The patient’s baseline characteristics and ovarian responses of the four PCOS phenotypes and control group are listed in Table 1. A significant difference did not exist in histories of spontaneous abortion and premature delivery, and the proportion of ICSI between the four PCOS phenotypes and control group (*P* = 0.922, *P* = 0.495, respectively). No significant differences in the type of infertility, fasting blood glucose (FBG), gonadotropin dose, and high-quality embryos between the four PCOS phenotypes were found. Significant differences were observed in age, BMI, SBP, DBP, duration of infertility, follicle-stimulating hormone, luteinizing hormone, total testosterone (To) concentration, anti-Müllerian hormone (AMH), antral follicle count (AFC), the number of follicles of diameter ≥ 14 mm and estradiol (E_2_) levels on the HCG trigger day, and the number of retrieved oocytes between the four PCOS phenotype and control groups (all *P* values were < 0.001).

**Table 1:** Baseline characteristics and ovarian responses of four PCOS phenotype and control groups.

Variables	Phenotype A (n = 452)	Phenotype B (n = 88)	Phenotype C (n = 119)	Phenotype D (n = 1228)	Control (n = 7016)	*P* values
Age (years)	28.89 ± 3.43^d,e^	29.53 ± 3.25^e^	28.43 ± 3.42^d,e^	29.56 ± 3.57^a,c,e^	30.97 ± 4.14^a,b,c,d^	< 0.001
BMI (kg/m^2^)	25.59 ± 3.74^d,e^	24.66 ± 3.54^e^	24.59 ± 3.66^e^	24.83 ± 3.67^a,e^	23.38 ± 3.33^a,b,c,d^	< 0.001
SBP	117.27 ± 11.88^d,e^	117.08 ± 12.42^e^	115.47 ± 12.95	114.62 ± 12.31^a,e^	113.1 ± 11.70^a,b,d^	< 0.001
DBP	71.16 ± 9.15^c,d,e^	70.53 ± 10.39	67.53 ± 9.47^a^	69.23 ± 9.25^a,e^	67.55 ± 8.85^a,d^	< 0.001
Duration of infertility	4.09 ± 2.33^e^	3.90 ± 2.66	3.55 ± 2.86	4.24 ± 2.66^e^	3.64 ± 2.62^a,d^	< 0.001
Type of infertility, n (%)						< 0.001
Primary	294/452 (65.0)^e^	54/88 (61.4)^e^	84/119 (70.6)^e^	758/1228 (61.7)^e^	3385/7016 (48.2)^a,b,c,d^	
Secondary	158/452 (35)^e^	34/88 (38.6)^e^	35/119 (29.4)^e^	470/1228 (38.3)^e^	3631/7016 (51.8)^a,b,c,d^	
History of spontaneous abortion (%)	52/452 (11.5)	11/88 (12.5)	11/119 (9.2)	143/1228 (11.6)	781/7016 (11.1)	0.922
History of premature delivery (%)	2/452 (0.4)	0/88 (0.0)	0/119 (0.0)	5/1228 (0.4)	15/7016 (0.2)	0.495
FBG (mmol/L)	5.26 ± 0.44	5.34 ± 0.45	5.30 ± 0.45	5.26 ± 0.45^e^	5.21 ± 0.44^d^	< 0.001
Basal FSH (IU/L)	5.62 ± 1.51^e^	6.01 ± 1.45^d^	5.75 ± 1.23^e^	5.54 ± 1.30^b,e^	6.31 ± 1.58^a,c,d^	< 0.001
Basal LH (IU/L)	11.83 ± 5.79^b,c,d,e^	8.13 ± 4.52^a,e^	8.40 ± 5.47^a,e^	8.19 ± 5.11^a,e^	5.12 ± 2.33^a,b,c,d^	< 0.001
Basal To (ng/dL)	62.50 ± 14.35^d,e^	60.46 ± 12.81^d,e^	61.66 ± 12.03^d,e^	30.88 ± 10.88^a,b,c,e^	24.64 ± 12.24^a,b,c,d^	< 0.001
AMH (ng/mL)	11.50 ± 4.18^b,c,d,e^	6.23 ± 3.46^a,c,d,e^	8.78 ± 4.29^a,b,e^	9.22 ± 4.15^a,b,e^	4.45 ± 2.76^a,b,c,d^	< 0.001
AFC	34.51 ± 12.03^b,c,d,e^	16.03 ± 3.57^a,c,d^	27.41 ± 9.22^a,b,e^	28.97 ± 8.28^a,b,e^	15.23 ± 5.89^a,c,d^	< 0.001
Gn dose (IU)	1791.77 ± 968.01	1940.91 ± 1009.77	1759.87 ± 814.84	1796.23 ± 907.69^e^	1892.88 ± 854.02^d^	0.001
No. of retrieved oocytes	19.04 ± 8.77^b,e^	15.23 ± 7.29^a,c,d^	18.85 ± 6.96^b,e^	17.91 ± 7.38^b,e^	13.47 ± 6.05^a,c,d^	< 0.001
ICSI (%)	128/324 (28.30)	28/60 (31.80)	42/77 (35.30)	370/858 (30.10)	2147/4869 (30.60)	0.647
No. of good quality embryos	5.36 ± 3.46^e^	5.19 ± 3.61	5.83 ± 3.52^e^	5.30 ± 3.39^e^	4.81 ± 3.06^a,c,d^	< 0.001

AMH, anti-Müllerian hormone; AFC, antral follicle count; BMI, body mass index; DBP, diastolic blood pressure; FBG = fasting blood glucose; FSH = follicle-stimulating hormone; Gn, gonadotropin; HCG, human chorionic gonadotropin; IU, international units; LH, luteinizing hormone; PCOS, polycystic ovary syndrome phenotype; SBP, systolic pressure; To, total testosterone concentration.

^a^*P* values < 0.05, compared with phenotype A.

^b^*P* values < 0.05, compared with phenotype B.

^c^*P* values < 0.05, compared with phenotype C.

^d^*P* values < 0.05, compared with phenotype D.

^e^*P* values < 0.05, compared with the control group.

### FET cycle characteristics and pregnancy outcome

Table 2 summaries FET cycle characteristics and pregnancy outcomes for the four PCOS phenotype and control groups. A difference was found for endometrial thickness and the presence of a corpus luteum between the five groups (*P* < 0.001 for both). Women with PCOS phenotype A showed an increased incidence of biochemical pregnancy, CP and premature delivery compared to those with PCOS phenotype D and in the control group (80.1% vs. 72.3% & 70.2%, *P* < 0.001; 71.0% vs. 64.9% & 62.9%, *P* = 0.005; 12.1% vs. 7.7% & 6.7%, *P* = 0.006, respectively), while the incidence of ectopic pregnancy and LB were comparable between the five groups (*P* = 0.596, *P* = 0.397, respectively). We also found a significantly higher abortion rate (20.6% & 19.2% vs 15.2%, *P* = 0.010) and lower ongoing pregnancy rate (78.5% & 80.4% vs. 83.9%, *P* = 0.023) among PCOS phenotypes A and D compared to control groups.

**Table 2:** FET cycle characteristics and pregnancy outcomes of four PCOS phenotype and control groups.

Variables	Phenotype A (n = 452)	Phenotype B (n = 88)	Phenotype C (n = 119)	Phenotype D (n = 1228)	Control (n = 7016)	*P* values
The presence of corpus luteum						< 0.001
Yes	86/452 (19.0) ^c,e^	20/88 (22.7) ^c,e^	62/119 (52.1) ^a,b,d,e^	273/1228 (22.2) ^c,e^	5004/7016 (71.3) ^a,b,c,d^	
No	366/452 (81.0) ^c,e^	68/88 (77.3) ^c,e^	57/119 (47.9) ^a,b,d,e^	955/1228 (77.8) ^c,e^	2012/7016 (28.7) ^a,b,c,d^	
No. of transferred blastocyst	1.03 ± 0.18	1.07 ± 0.25	1.04 ± 0.20	1.03 ± 0.18	1.03 ± 0.17	0.637
Days of embryos frozen	101.24 ± 63.59^b,e^	128.41 ± 110.43^a,d,e^	109.23 ± 76.06	102.88 ± 64.56^b,e^	111.98 ± 78.74^a,b,d^	< 0.001
Endometrial thickness in FET (mm)	9.1 ± 1.4^c,e^	9.5 ± 1.9	9.6 ± 1.5^a,d^	9.2 ± 1.4^c,e^	9.6 ± 1.7^a,d^	< 0.001
Biochemical pregnancy (%)	362/452 (80.1)^d,e^	63/88 (71.6)	91/119 (76.5)	888/1228 (72.3)^a^	4924/7016 (70.2)^a^	< 0.001
CP (%)	321/452 (71.0)^d,e^	53/88 (60.2)	82/119 (68.9)	797/1228 (64.9)^a^	4416/7016 (62.9)^a^	0.005
Ectopic pregnancy (%)	3/321 (0.9)	0/53 (0.0)	0/82 (0.0)	3/723 (0.4)	40/4416 (0.9)	0.596
Abortion (%)	66/321 (20.6)^e^	9/53 (17.0)	12/82 (14.6)	153/797 (19.2)^e^	670/4416 (15.2)^a,d^	0.010
Ongoing pregnancy (%)	252/321 (78.5)^e^	44/53 (83)	70/82 (85.4)	641/797 (80.4)^e^	3706/4416 (83.9)^a,d^	0.023
Premature delivery (%)	39/321 (12.1)^d,e^	5/53 (9.4)	8/82 (9.8)	61/797 (7.7)^a^	295/4416 (6.7)^a^	0.006
LB (%)	252/452 (55.8)	44/88 (50.0)	70/119 (58.8)	638/1228 (52.0)	3687/7016 (52.6)	0.397

The presence or absence of a corpus luteum was based on endometrial preparation protocols; that is, natural and ovulation induction cycles were considered to mean the formation of a corpus luteum, and a hormone replacement therapy cycle was regarded as meaning the absence of a corpus luteum.

CP, clinical pregnancy; FET, frozen-thawed embryo transfer; LB, live birth.

^a^*P* values < 0.05, compared with phenotype A.

^b^*P* values < 0.05, compared with phenotype B.

^c^*P* values < 0.05, compared with phenotype C.

^d^*P* values < 0.05, compared with phenotype D.

^e^*P* values < 0.05, compared with the control group.

Potential confounders of abortion included those variables (included age, BMI, duration and type of infertility, FBG, the number of retrieved oocytes and good quality embryos, days of embryos frozen, and the presence or absence of a corpus luteum) which *P* < 0.05 in univariate logistic regression analysis and endometrial thickness in FET. Potential confounders of premature delivery (*P* < 0.05 in univariate logistic regression analysis) included SBP, DBP, BMI, type of infertility, To, AMH, AFC, and the number of transferred blastocysts in FET. After adjusting for potential confounders, PCOS phenotypes A and D (vs. control) were associated with an elevated risk of abortion (adjusted OR, 1.476, 95% CI, 1.077–2.024, *P* = 0.016; adjusted OR, 1.348, 95% CI, 1.080–1.682, *P* = 0.008, respectively). PCOS phenotype A (vs. control) was not a significant risk factor for preterm delivery after adjusting results for potential confounders (*P* = 0.144; Table 3).

**Table 3:** Crude and adjusted ORs of various PCOS phenotypes for abortion and premature delivery.

	Crude OR (95% CI)	*P* values	Adjusted OR (95% CI)	*P* values
**Abortion (%)**				
PCOS phenotype		0.010		0.022
PCOS-A	1.447 (1.091–1.920)	0.010	1.474 (1.072–2.026)	0.017
PCOS-B	1.144 (0.556–2.354)	0.716	1.122 (0.537–2.345)	0.759
PCOS-C	0.958 (0.517–1.778)	0.893	1.213 (0.644–2.285)	0.550
PCOS-D	1.328 (1.094–1.614)	0.004	1.413 (1.123–1.777)	0.003
**Premature delivery (%)**				
PCOS phenotype		0.006		0.683
PCOS-A	1.932 (1.355–2.756)	< 0.001	1.499 (0.871–2.578)	0.144
PCOS-B	1.455 (0.575–3.683)	0.429	1.178 (0.440–3.152)	0.745
PCOS-C	1.510 (0.721–3.162)	0.274	1.271 (0.558–2.894)	0.569
PCOS-D	1.158 (0.869–1.542)	0.316	1.053 (0.737–1.504)	0.776

CI, confidence interval; OR, odds ratio; PCOS, polycystic ovary syndrome.

## Discussion

This study revealed a marked incidence of increased abortion in women of PCOS phenotype A and D groups after controlling for potential confounders. However, a finding of an elevated risk of premature delivery for the PCOS phenotype A group did not occur after controlling for potential confounders.

Abortion is a common complication of pregnancy. The etiology of abortion is complex: obesity^20^, insulin resistance^21^, hyperandrogenism^22^, the quality of oocytes, and endometrial abnormalities might be associated with the occurrence of abortion^23,24^. As reported in prior studies, women with PCOS are associated with an increased risk of abortion^25,26^. In addition, the incidence of abortion was increased in women who underwent ART^27^, with a possible mechanism related to corpus luteum insufficiency^28^.

In our study, the incidence of abortion was significantly increased in PCOS phenotypes A and D, and with the coexistence of OA and PCOM in PCOS phenotypes A and D. We speculate that the combination of OA and PCOM might increase the risk of abortion by affecting oocyte quality. Oocyte maturation and the fertility rate of anovulatory women were significantly lower than those of regular cycling women^29^; their embryo development ratio followed a similar trend^30^. A study of anovulation in cows found that anovulation also leads to major shifts in gene expression in elongated conceptuses during preimplantation stages; transcripts involved with the control of energy metabolism and DNA repair were downregulated, whereas genes linked to apoptosis and autophagy were upregulated^31^. Furthermore, a recent study revealed decreased oocyte quality in PCOM due to the abnormal activation of one-carbon metabolism and hypermethylation of mitochondrial DNA^32^. These results support the above conjecture.

Moreover, we also observed that the rate for the presence of a corpus luteum (because of different endometrial preparation protocols for FET) in PCOS phenotypes A and D was significantly lower than that in controls, which might be another reason for the higher rate of abortion. In clinical practice, for PCOS women with OA, clinicians generally adopt a hormone replacement therapy (HRT) cycle to prepare the endometrium for FET^33^. Recently, Xu et al.^34^ noticed that HRT cycles were related to a higher abortion rate, which is consistent with the results of a prior study^35^. The corpus luteum is an important source of hormones in pregnant women^12,36^, but during endometrial preparation, a corpus luteum is absent in an HRT cycle. Additionally, administering exogenous hormone in an HRT cycle might increase the risk of thromboembolic events and could damage placentation, which may then lead to abortion^37,38^.

It is generally known that obesity has an undesirable impact on women’s reproduction^39^. Obesity increases the rate of abortion^40^ and is an independent risk factor for abortion^41^. Obesity affects follicle development by affecting sex hormone secretion and metabolism^39^; other studies have found adverse effects of obesity on the quality of the embryo^42^ and endometrial receptivity^43^. In the present study, the BMI in PCOS phenotype A and D groups was significantly higher than that in the control group. Before the initiation of FET, obese women can reduce their weight to optimize pregnancy outcomes^44^. Interestingly, we also noticed that the age of the women with PCOS phenotypes A and D was lower than that of women in the control group. It is well documented that maternal age increases the incidence of abortion^45,46^. It is possible that other factors masked the effect of age on abortion. The average age of patients was 30.62 ± 4.07 years in our study, which may have a relatively small impact on abortion. Previous studies found that an advanced age increased the risk of abortion, usually over the age of 35 or 38^47,48^. In addition, previous studies have suggested that HA is associated with increased rates of abortion^49^. However, this was not found in our study. A recent systematic review and meta-analysis also showed that HA does not increase the risk of abortion in patients with PCOS^21^.

In this study, we found, for the first time, that women with PCOS phenotypes A and D had an elevated risk of abortion after FET. We speculated that this was associated with the coexistence of OA and PCOM by affecting the quality of oocytes and the formation of a corpus luteum. Therefore, for women with PCOS phenotypes A and D, lifestyle interventions such as improved diet and increased exercise were used to reduce body weight before FET; natural or ovulation-induced cycles are recommended as a priority for endometrial preparation in FET, and an appropriately increased luteal phase support. Additionally, pregnancy follow-up after obtaining a clinical pregnancy should be strengthened, and, with any sign of an abortion, treatment should be promptly provided.

However, the investigation of several risk factors, such as environmental factors, physical activities, and social factors, could not be included in this study. Due to the retrospective nature of this study, data on insulin resistance is lacking, and so was not included in the analysis. In addition, although we have the advantage of large sample size, due to the characteristics of the Chinese women, a large difference in sample size between various phenotype groups was observed. Therefore, a further, large-scale, and rigorous prospective validation study is necessary for the future.

## Conclusions

In summary, our findings demonstrate women with PCOS phenotypes A and D had an elevated risk of abortion after FET, which might be associated with the coexistence of OA and PCOM by affecting the quality of oocytes and the formation of a corpus luteum. It is suggested that when performing FET, clinicians should individually manage women with PCOS phenotypes A and D to reduce the rate of abortion and increase the rate of LB, and achieve better pregnancy outcomes.

## Data Availability

The data and materials are available from the corresponding author on reasonable requests.
